# Exercising self-control increases responsivity to hedonic and eudaimonic rewards

**DOI:** 10.1093/scan/nsaf016

**Published:** 2025-01-30

**Authors:** Chengli Huang, Zhiwei Zhou, Douglas J Angus, Constantine Sedikides, Nicholas J Kelley

**Affiliations:** Centre for Research on Self and Identity, School of Psychology, University of Southampton, Southampton SO17 1BJ, United Kingdom; Centre for Research on Self and Identity, School of Psychology, University of Southampton, Southampton SO17 1BJ, United Kingdom; School of Psychology, Bond University, Gold Coast 4229, Australia; Centre for Research on Self and Identity, School of Psychology, University of Southampton, Southampton SO17 1BJ, United Kingdom; Centre for Research on Self and Identity, School of Psychology, University of Southampton, Southampton SO17 1BJ, United Kingdom

**Keywords:** self-control, effort, reward positivity, hedonic rewards, eudaimonic rewards

## Abstract

The reward responsivity hypothesis of self-control proposes that irrespective of self-control success, exercising self-control is aversive and engenders negative affect. To countermand this discomfort, reward-seeking behavior may be amplified after bouts of self-control, bringing individuals back to a mildly positive baseline state. Previous studies indicated that effort—an integral component of self-control—can increase reward responsivity. We sought to test and extend the reward responsivity hypothesis by asking if exercising self-control increases a neural marker of reward responsivity [Reward Positivity (RewP)] differentially for hedonic rewards or eudaimonic rewards. We instructed participants (*N* = 114) to complete a speeded reaction time task where they exercised self-control (incongruent Stroop trials) or not (congruent Stroop trials) and then had the opportunity to win money for themselves (hedonic rewards) or a charity (eudaimonic rewards) while electroencephalography was recorded. Consistent with the reward responsivity hypothesis, participants evinced a larger RewP after exercising self-control (vs. not exercising self-control). Participants also showed a larger RewP for hedonic over eudaimonic rewards. Self-control and reward type did not interactively modulate RewP, suggesting that self-control increases reward responsivity in a domain-general manner. The findings provide a neurophysiological mechanism for the reward responsivity hypothesis of self-control and promise to revitalize the relevant literature.

## Introduction

The ability to override or alter motivated responses (i.e. self-control) is crucial for goal-directed behavior and contributes to many consequential outcomes including physical health, psychological well-being, ethical decision-making, and successful interpersonal relationships (Vohs and Baumeister 2016). Conversely, failures in self-control have negative consequences in these and other domains. Self-control has thus been of keen interest to psychologists, neuroscientists, philosophers, and the public. The most influential model of self-control, the resource model (Baumeister et al. 1998), though generative, has come under intense scrutiny in recent years (Carter and McCullough 2014, Carter et al. 2015, MS et al. 2016, Vohs et al. 2021). In response to empirical challenges to this model, the reward responsivity hypothesis of self-control proposes that exercising self-control does not influence behavior generally but influences the reward system specifically (Kelley et al. 2019). The purpose of this study is to (I) examine the neural basis of the reward responsivity hypothesis of self-control by assessing how self-control exertion impacts the Reward Positivity (RewP) and (II) expand this hypothesis by testing the extent to which exercising self-control influences the reward system differently for hedonic versus eudaimonic rewards.

### The resource model of self-control

Self-control has been extensively investigated through the lens of the resource model (Baumeister et al. 1998). For 30 years, this model has enjoyed widespread influence in social/personality psychology and psychological science in general. According to it, the capacity to override or alter one’s responses depends on limited inner resources or strength (Baumeister et al. 1998, 2007). Acts of self-control are theorized to consume (i.e. deplete) this strength, resulting in a temporary decline in the capacity for self-control (i.e. ego depletion). In support, numerous studies have found that engaging in a taxing (or depleting) self-control task undermines performance on subsequent demanding tasks (Baumeister et al. 2007, 2018, 2023). Mechanistically, these effects were thought to be driven by glucose (Gailliot et al. 2007), although meta-analyses have cast doubt on this interpretation (Dang 2016).

Nevertheless, empirical challenges, controversies, and debates related to the validity of the resource model have arisen. An initial meta-analysis of the relevant literature reported evidence for consistent and large effects (Hagger et al. 2010), but more recent meta-analyses have concluded that the effect is negligible after adjusting for publication bias (Carter and McCullough 2014, Carter et al. 2015). Multi-laboratory experiments obtained nonsignificant aftereffects of self-control exertion (MS et al. 2016, Vohs et al. 2021), whereas other preregistered experiments obtained statistically significant, albeit smaller than expected effects (Dang et al. 2017, Garrison et al. 2019). Collectively, the mechanisms and aftereffects of self-control exertion remain poorly understood.

### Reward responsivity hypothesis of self-control

The reward responsivity hypothesis of self-control (Kelley et al. 2019) was a response to controversies and challenges to the resource model. According to this hypothesis, irrespective of self-control success, exercising self-control is aversive and engenders negative affect (Kurzban 2016, David et al. 2024). To countermand this discomfort, reward-seeking behavior may be augmented after bouts of self-control, bringing individuals back to a mildly positive baseline state. In other words, the reward responsivity hypothesis of self-control states that exercising self-control does not influence behavior generally, but it influences specifically the reward system (Kelley et al. 2019). The latter aligns with the core tenet of the process model of self-control, which suggests that self-control shifts attention and motivation toward rewards (Inzlicht et al. 2014). In contrast, the resource model does not explicitly predict that exercising self-control increases subsequent reward-related impulse strength. Instead, it posits that engaging in taxing self-control tasks depletes limited resources, leading to impaired performance on subsequent demanding tasks in general. Yet, several studies inspired by the resource model have reported evidence that exercising self-control increases subsequent reward-seeking behavior, including eating, spending, and sexual behavior (Baumeister et al. 2007). These behavioral outcomes could be due to a reduction in the capacity for self-control (as the resource model initially assumed) or increases in reward responsivity (as the reward responsivity hypothesis proposed). Several studies in line with the reward responsivity hypothesis of self-control have circumvented this interpretational ambiguity by instructing participants to complete reward-related tasks requiring little to no self-control. For example, Finley and Schmeichel (2019) observed that self-control exertion enhances self-reported approach motivation and positive emotional reactivity. Our primary goal here was to examine whether exercising self-control would enhance a neural marker of reward responsivity: an Event-Related Potential (ERP), known as the Reward Positivity (RewP).

### Self-control and RewP

The RewP (Carlson et al. 2011, Foti et al. 2011, Walsh and Anderson 2012) is sensitive to feedback signaling the outcome of an action. The RewP peaks ∼200–300 ms after feedback onset (Glazer et al. 2018), is most pronounced over fronto-central sites (Miltner et al. 1997, Holroyd et al. 2008, 2011), and is modulated by the delivery of advantageous versus neutral or disadvantageous outcomes (Ma et al. 2014, San Martín et al. 2016, Harmon-Jones et al. 2020a, 2020b, Luo et al. 2022). The RewP is partly driven by activity in reward-related subcortical regions such as the striatum (Carlson et al. 2011, 2015, Foti et al. 2011, 2014, Becker et al. 2014).

We conceptualize “effort” as the mobilization of general resources—both mental and physical—to execute behavior (Gendolla and Wright 2009). It involves the allocation of energy toward achieving any goal requiring energy, regardless of whether self-control is needed. Therefore, self-control is a specific form of effort that entails overriding impulses and resisting temptation. [Several studies have operationalized self-control as effort (e.g. “How much effort did you exert on …?”; Muraven et al. 1998, 2006).] In fact, training in effort enhances general self-control capacity (for a review, see Smith et al. 2019). Moreover, effort constitutes an integral component of self-control and can determine self-control behavior (Kotabe and Hofmann 2015). Convergent evidence indicates that effort increases the RewP. For example, Pan et al. (2023) found that higher effort conditions evoke greater RewP neural amplitude response. Similarly, Bogdanov et al. (2022) reported that the RewP is significantly elevated in trials requiring more versus less cognitive effort. Furthermore, Ma et al. (2014) demonstrated that demanding mental arithmetic problems, but not simpler ones, are associated with larger RewP amplitudes. Similarly, Harmon-Jones et al. (2024), using an effortful task-switching paradigm, observed that high effort, compared to low effort, yields a larger RewP amplitude when participants believe that their effort led to the reward. These findings were corroborated by self-reports, where self-reported effort exertion was associated with larger RewP differences (Harmon-Jones et al. 2020a, 2020b). In summary, the literature suggests that effort exertion modules the RewP. Given that effort constitutes an integral component of self-control (Kotabe and Hofmann 2015), we hypothesized that exerting self-control would enhance the RewP.

Rewards can take many forms. One of the earliest and most enduring conceptualizations of rewards distinguishes between hedonic and eudaimonic ones. Hedonic rewards are defined in terms of pleasure and comfort, whereas eudaimonic rewards are defined in terms of meaning and self-realization (Ryan and Deci 2001, Huta and Waterman 2014, Telzer et al. 2014). Thus, hedonic rewards are very pleasurable and self-focused, such as enjoying material goods and playing video games, whereas eudaimonic rewards are intrinsically meaningful and purposeful, such as helping strangers and donating to charity (Shizgal 1999, Steger et al. 2008a, Telzer et al. 2014). Although hedonism and eudaimonia are positively associated (Kashdan et al. 2008, Disabato et al. 2016, Goodman et al. 2018), a good deal of studies highlight their relative independence and differentiation (Gallagher et al. 2009, Henderson et al. 2013, Huta and Waterman 2014, Joshanloo 2016). Neural activity associated with eudaimonic rewards (e.g. donating money to family) predicts increases in well-being, whereas neural activity associated with hedonic rewards (e.g. keeping money for oneself) predicts decreases in well-being (Telzer et al. 2014, Luo et al. 2019, 2022). Crucially, some recent studies indicate that hedonic and eudaimonic rewards also influence reward responsivity differently, although the findings are inconsistent. For instance, one study reported that hedonic rewards (i.e. winning rewards for oneself) elicited a larger RewP difference wave compared to eudaimonic rewards (i.e. winning rewards for charity; Luo et al. 2019). However, other studies found comparable RewP amplitudes between hedonic rewards and eudaimonic rewards (Luo et al. 2022, Zhang et al. 2023). The inconsistent findings highlight the need to clarify the distinct neural processes underlying these different forms of reward. Thus, our secondary goal was to examine whether the effects of self-control exertion on the RewP would differ for hedonic versus eudaimonic rewards.

### Overview

Research and theory indicate that exercising self-control enhances the RewP. However, it is unclear whether this effect occurs for hedonic rewards, eudaimonic rewards, or both. On the one hand, exercising self-control may increase hedonic reward responsivity. After all, the majority of studies examining the effects of self-control exertion on reward responsivity have focused on hedonic rewards (Kelley et al. 2019), and hedonic rewards (vs. rewards for others) more strongly activate the ventral striatum (Morelli et al. 2015), which is a neural generator of the RewP (Carlson et al. 2011). On the other hand, self-control may increase eudaimonic reward responsivity. In support of this view, recent research suggests that effort exertion increases meaning in life (Campbell et al. 2024). Insofar as meaning is more strongly tied to eudaimonic than hedonic rewards, exercising self-control may increase the RewP moreso for eudaimonic rewards. Still, another option is that exercising self-control increases the RewP similarly for hedonic and eudaimonic rewards. Such a perspective is consistent with the strong links between the two types of rewards (Kashdan et al. 2008, Disabato et al. 2016, Goodman et al. 2018) and the common neural processes across them (Liu et al. 2011, Sescousse et al. 2013, Morelli et al. 2015). To test these competing viewpoints, participants exerted self-control (incongruent Stroop trials) or not (congruent Stroop trials) in a speeded reaction time task where they had the opportunity to win money for themselves (a hedonic reward) or a charity of their choosing (a eudaimonic reward) while electroencephalography (EEG) was recorded. We measured participants’ reward responsivity via the RewP.

## Materials and methods

### Participants and design

Following past research on the RewP to hedonic and eudaimonic rewards (Luo et al. 2019, 2022), we used G*Power (Faul et al. 2009) assuming a small effect size (Cohen’s *f* = 0.10), six measures (deriving from a 2 × 3 within-subjects design), *α* = 0.05, power (1 − *β*) = 0.80, and a moderate relation among repeated measures (*r* = 0.50). Based on these parameters, 109 participants were required. We oversampled assuming data loss and recruited 121 participants from the University of Southamptom psychology participant pool in exchange for course credit and task winnings. We tested them in private cubicles and via computer. We excluded seven participants from EEG analyses because >50% of their trials had been rejected due to artifacts or wrong response, leaving insufficient (<30) trials, and thus failing to meet the requirement for ERP analysis (Cai et al. 2016). The final sample comprised 114 participants (93 women, 18 men, and 3 nonbinary), aged between 18 and 37 years (*M* = 19.63, s.d. = 2.99). We did not collect ethnicity information, but >90% of the University of Southampton undergraduates are White. The experimental protocol was approved by the Ethics Committee of the University of Southampton (No. 79802). We used a 3 (reward: hedonic, eudaimonic, control) × 2 (congruency: congruent, incongruent) within-subjects design. We addressed the issue of multiple comparisons using Bonferroni corrections.

### Procedure

**Figure 1.**
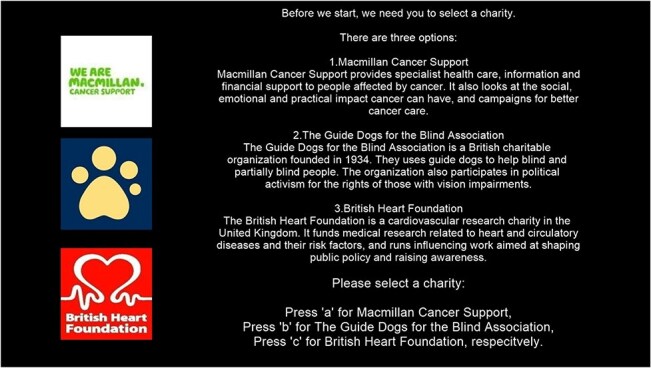
Charity target choice prior to the MID task.

*Notes*. We described the function of each charity and how they use donations. We took descriptions from Wikipedia and edited them down to 35–40 words.

All participants were familiarized with the electrophysiology laboratory and EEG recording procedure before providing informed consent. Participants were then fitted with recording electrodes and seated in a comfortable armchair ∼80 cm away from a 60 cm × 33.5 cm monitor in a quiet laboratory room. They engaged in two core assessments: an 8-min resting-state EEG session (as part of a different project) and a modified monetary incentive delay (MID) task (Knutson et al. 2001). Following Luo et al. (2022), participants first read a brief description of three representative charities: Macmillan Cancer Support, Guide Dogs for the Blind Association, and British Heart Foundation (Fig. 1). Subsequently, they chose one of the three charities as the donation target. In the hedonic condition, the money they won belonged to them, whereas, in the eudaimonic condition, the money they won belonged to their chosen target.

**Figure 2.**
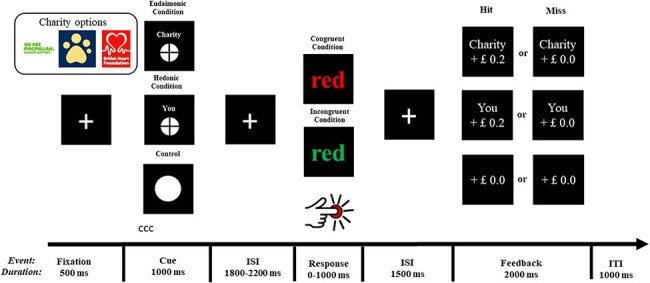
Trial structure of the MID task.

*Note*. ISI, inter-stimulus interval; ITI, inter-trial interval.

We report the trial structure in Fig. 2. Each trial began with a 500-ms fixation-cross in the center of the screen. Thereafter, we presented participants with an incentive cue for 1000 ms. There were three cue types in each session that prompted the object of the win money: self (i.e. hedonic condition), charity (i.e. eudaimonic condition), and nobody (i.e. neutral control condition). In the hedonic condition (signaled by a circle with a cross inside labeled with “You” above), we informed participants of the potential monetary win for themselves. In the eudaimonic condition (signaled by a circle with a cross inside labeled with “Charity” above), we informed participants of the potential monetary win for the charity. In the neutral control condition (signaled by a circle), we informed participants that they would win money neither for themselves nor for the charity regardless of their efforts. We presented these cues with equal probability and in a random order. We followed the cue with a fixation cross appearing 1800–2200 ms. Then, we presented participants with the target stimulus, a color word with either a congruent (i.e. congruent trials) or an incongruent (i.e. incongruent trials) ink color. We instructed them to ignore the meaning of the word and identify the ink color of the word as quickly and concretely as possible with their dominant hand by pressing the keyboard. We presented each word stimulus on the screen until a response (key-pressing) occurred, but no longer than 1000 ms. Lastly, after a 1500 ms fixation-cross, we signaled the outcome of each trial by feedback stimulus presented for 2000 ms. There were two types of feedback in each condition. In the hedonic condition, the feedback of “Self + £0.2” would be present if the response were correct and fast enough; otherwise, the feedback would be “Self + £0.0.” In the eudaimonic condition, the feedback of “Charity + £0.2” would be present if the response were correct and fast enough; otherwise, the feedback would be “Charity + £0.0.” In the neutral control condition, the feedback would always be “+ £0.0” regardless of the response.

Participants completed a practice block of 27 trials prior to the experimental blocks to allow them to learn the association between each cue and experimental condition. The experiment consisted of 324 trials and was divided into six blocks of 54 trials. Each block involved a randomized distribution of three conditions. Participants received a self-paced break after each block. We programmed and administered the experiment using PsychoPy (Version 2021.2.3; Peirce 2007). At the end of the study, we compensated participants with £10 ($12.77, €11.69, or ¥91.51) (in addition to course credits and irrespective of task performance) and gave them the donation website for each of three charities.

### Data recording and data analysis

We collected the EEG data continuously from 64 scalp sites using Ag/AgCl electrodes mounted in an elastic cap (Neuroscan, NC), with an online reference to the left mastoid and an off-line algebraic rereference to the average of left and right mastoids. We mounted a ground electrode midway between FPz and Fz. We recorded the vertical electrooculogram and horizontal electrooculogram from two pairs of electrodes, with one placed above and below the left eye, and another placed 10 mm from the outer canthi of each eye. We based the electrode cap on the 10–20 system. We kept electrode impedances below 5 kΩ. Also, we amplified and sampled the signals at 1000 Hz with an online bandpass filter from 0.10 to 100 Hz.

In offline processing, we initially preprocessed the EEG data by using EEGLAB, an open-source toolbox running in the MATLAB environment (Delorme and Makeig 2004). We digitally filtered the EEG data with a band-pass filter (high pass: 0.10 Hz, low pass: 40 Hz, 50 Hz notch), segmented them from 200 ms prior to 800 ms following the onset of feedback, and baseline corrected them to the −200 to 0 ms. We identified bad channels by visual inspection of the waveforms and replaced them by using a spherical spline identified interpolation (Perrin et al. 1989). We corrected segments contaminated by blinks, eye movements, and other artifacts using an independent component analysis (ICA) algorithm (Delorme and Makeig 2004) and ICLabel, a proposed statistical model, to automatically label ICA components (Pion-Tonachini et al. 2019). We also excluded bad segments where a voltage deviation on any channel is ±100 μV. Finally, we used extracted average waveforms for each participant and condition to calculate grand average waveforms.

Following best practices (i.e. to employ multiple comparisons correction, to average across the electrode sites, and to use difference scores, that is, RewP difference wave; Luck and Gaspelin 2017), previous studies (Harmon-Jones et al. 2020b, Luo et al. 2022), and inspection of the grand average waveforms, we quantified the RewP as the mean amplitude on a 100-ms window (i.e. 280–380 ms) after feedback onset over frontal-central sites (i.e. Fz, FCz, and Cz). Also, we calculated the RewP difference wave as the difference between the ERP response to gains (i.e. rewards) and the ERP response to neutral (Ma et al. 2014, Luo et al. 2019). [In previous studies, the RewP effect was calculated as the difference between the ERP in response to gains and the ERP in response to loss (San Martín et al. 2016, Harmon-Jones et al. 2020a, Luo et al. 2022, 2024) or between the ERP in response to gains and the ERP in response to neutral (Ma et al. 2014, Luo et al. 2019). However, we included no loss condition (i.e. a condition in which participants would lose money) in the current study. Considering that prior work has found that ERPs to neutral feedback and loss feedback are equivalent in this type of task (Holroyd et al. 2006, Experiment 5), we calculated the RewP effect as the difference between the ERP in response to gains and the ERP in response to neutral.]

## Results

### Hit rate and reaction time

We excluded data from trials where participants provided an improper response (<200 ms). All participants’ mean hit rate and reaction time were within 3 s.d. from the mean. We conducted a 3 (reward: hedonic, eudaimonic, control) × 2 (congruency: congruent, incongruent) repeated analysis of variance (ANOVA) on hit rate and reaction time. The main effects of congruency were significant, as participants had a higher hit rate, *F*(1, 113) = 166.79, *P* < .001, *ƞ*_p_^2^ = 0.60, and were faster, *F*(1, 113) = 372.61, *P* < .001, *ƞ*_p_^2^ = 0.77, on congruent than incongruent trials. The main effects of reward were significant for both hit rate, *F*(2, 112) = 9.08, *P* < .001, *ƞ*_p_^2^ = 0.14, and reaction time *F*(2, 112) = 15.59, *P* < .001, *ƞ*_p_^2^ = 0.22. Compared to control trials, participants had a higher hit rate on hedonic trials (*P* < .001) and tended to have a higher hit rate on eudaimonic trials (*P* = .092); also, participants had a higher hit rate on hedonic than eudaimonic trials (*P* = .007). The pattern was similar for reaction time: compared to control trials, participants were faster on hedonic (*P* < .001) and eudaimonic (*P* = .001) trials, and they were faster on hedonic than eudaimonic trials (*P* = .017).

The reward × congruency interactions were significant for both hit rate, *F*(2, 112) = 4.88, *P* = .009, *ƞ*_p_^2^ = 0.08, and reaction time, *F*(2, 112) = 38.65, *P* < .001, *ƞ*_p_^2^ = 0.41. Hit rates were higher and reaction times were shorter for congruent versus incongruent trials for each reward type (*P *< .001). Differences between congruent and incongruent trials were largest for eudaimonic reward trials compared to hedonic reward trials and control trials (hit rate: *d*_Eudaimonic_ = 0.96, *d*_Hedonic_= 0.84, *d*_Control_ = 0.84; reaction time: *d*_Eudaimonic_ = 2.38, *d*_Hedonic_= 1.65, *d*_Control_ = 1.43). We reported means and standard deviations in Table 1.

**Table 1.** Means and standard deviations for hit rate, reaction time, RewP, and RewP difference wave.

Hit rate				
	Eudaimonic	Hedonic	Control	Average
Incongruent	43.87 (6.05)	45.29 (5.68)	43.86 (6.63)	44.34 (5.43)
Congruent	50.12 (3.29)	50.28 (3.18)	48.72 (4.39)	49.71 (3.12)
Average	47.79 (3.83)	47.70 (4.00)	46.29 (4.98)	
**Reaction time (ms)**				
	Eudaimonic	Hedonic	Control	Average
Incongruent	674.04 (65.52)	658.55 (66.37)	669.66 (66.92)	667.42 (63.39)
Congruent	594.04 (54.78)	596.87 (54.47)	616.74 (57.39)	602.55 (52.97)
Average	634.04 (57.26)	627.71 (56.77)	643.20 (58.88)	
**RewP (μV)**				
	Eudaimonic	Hedonic	Control	Average
Incongruent	5.35 (4.70)	6.24 (4.68)	4.66 (4.60)	5.42 (4.66)
Congruent	4.16 (4.38)	5.29 (4.63)	4.12 (4.40)	4.53 (4.47)
Average	4.76 (4.54)	5.77 (4.66)	4.39 (4.50)	
**RewP difference wave (μV)**				
	Eudaimonic-control	Hedonic-control	Average	
Incongruent	0.69 (2.94)	1.58 (2.96)	5.42 (4.66)	
Congruent	0.05 (3.16)	1.17 (3.04)	4.53 (4.47)	

### The RewP

**Figure 3.**
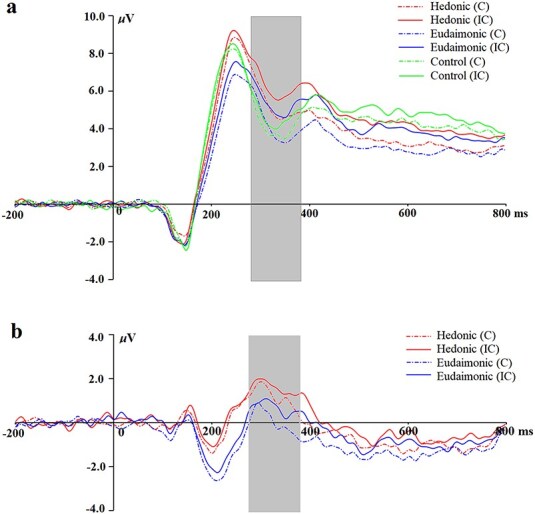
Grand average event-related potential waveforms of the RewP as a function of self-control exertion.

*Notes*: (a) Grand averages of RewP for all conditions. (b) RewP difference waves (Reward − Control) in hedonic and eudaimonic conditions. The RewP measurement window (i.e. 280–380 ms) shaded in light gray. C = congruent trials; IC = incongruent trials.

**Figure 4.**
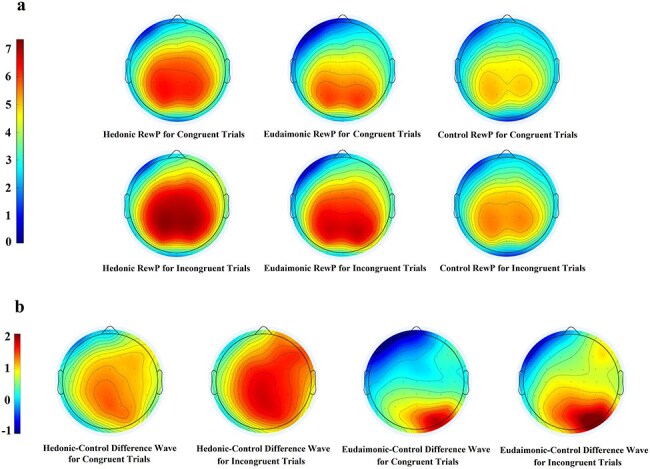
Topographical maps of the RewP as a function of self-control exertion.

*Notes*. (a) Topographical maps of the RewP for all conditions. (b) Topographical maps of the RewP difference waves (Reward − Control) in hedonic and eudaimonic conditions.

We conducted a 3 (reward: hedonic, eudaimonic, control) × 2 (congruency: congruent, incongruent) repeated measures ANOVA on RewP amplitudes. We obtained a significant main effect of reward, *F*(2, 112) = 19.09, *P* < .001, *ƞ*_p_^2^ = 0.25. *Post hoc* analysis showed that the RewP was larger on hedonic (*M* = 5.77, s.d. = 4.66) than on eudaimonic (*M* = 4.76, s.d. = 4.54, *P* < .001) trials, and higher than in the control (*M* = 4.39, s.d. = 4.50, *P* < .001) trials. However, there was no significant difference on RewP between eudaimonic trials and control trials (*P* = .396). In addition, consistent with the reward responsivity hypothesis of self-control (Kelley et al. 2019), the RewP was larger after self-control was exerted (i.e. incongruent trials, *M* = 5.42, s.d. = 4.66) compared to not exerted (i.e. congruent trials, *M* = 4.53, s.d. = 4.47), *F*(1, 113) = 42.04, *P* < .001, *ƞ*_p_^2^ = 0.27. The interaction was not significant, *F*(2, 112) = 2.25, *P* = .110, *ƞ*_p_^2^ = 0.04. We reported means and standard deviations of RewP amplitudes in Table 1. We depicted grand average waveforms in Fig. 3 and Supplementary Fig. S1, and the corresponding topographic maps in Fig. 4.

Next, we examined the modulation of the RewP difference wave using a 2 (reward: hedonic, eudaimonic) × 2 (congruency: congruent, incongruent) repeated measures ANOVA. Consistent with past research (Luo et al. 2019), the RewP difference wave was larger on hedonic (*M* = 1.42, s.d. = 3.00) than on eudaimonic (*M* = 0.38, s.d. = 3.05) trials, *F*(1, 113) = 22.50, *P* < .001, *ƞ*_p_^2^ = 0.17. In addition, consistent with the reward responsivity hypothesis of self-control (Kelley et al. 2019), the RewP difference wave was larger after self-control was exerted (i.e. incongruent trials, *M* = 1.17 s.d. = 3.10) compared to not exerted (i.e. congruent trials, *M* = 0.63, s.d. = 2.95), *F*(1, 113) = 4.37, *P* = .039, *ƞ*_p_^2^ = 0.04. However, the interaction was not significant, *F*(1, 113) = 0.61, *P* = .438, *ƞ*_p_^2^ = 0.01. We reported means and standard deviations of the RewP difference wave in Table 1. We depicted grand average waveforms in Fig. 3 and Supplementary Fig. S2, and the corresponding topographic maps in Fig. 4.

## Discussion

We aimed to provide a rigorous test of the reward responsivity hypothesis of self-control (Kelley et al. 2019). Although this hypothesis is agnostic about how self-control exertion influences different types of rewards (hedonic vs. eudaimonic), it implicitly suggests that exercising self-control enhances reward responsivity generally. However, the majority of the literature on self-control and reward responsivity has focused on hedonic rewards such as responsivity to food (Hofmann et al. 2007, Vohs et al. 2011, Study 3, Imhoff et al. 2014, Haynes et al. 2016), drugs (Muraven et al. 2002, Shmueli and Prochaska 2009, Christiansen et al. 2012, Schlauch et al. 2015), and money (Bruyneel et al. 2009, Schmeichel et al. 2010, Study 2b, Achtziger et al. 2015, Osgood and Muraven 2015). Given this and recent evidence that exercising self-control increases meaning in life (Campbell et al. 2024), we sought to examine whether reward type (hedonic vs. eudaimonic) moderates the effect of self-control on reward responsivity. Consistent with the reward responsivity hypothesis of self-control, we showed that exercising self-control increases immediate neural responsivity to rewards (as indexed by RewP) in a domain-general fashion.

### Theoretical implications

The findings are consistent with theorizing in the self-control literature. According to the process model of self-control, exercising self-control causes shifts in attention and motivation toward rewards and gratification (Inzlicht and Schmeichel 2012, Inzlicht et al. 2014). Insofar as the RewP is a reward prediction error linked to motivation and attention (Lange et al. 2012, Threadgill and Gable 2016), its enhancement following self-control exertion is consistent with the central premise of the process model of self-control. Our results are also interpretable through the lens of the integrative self-control theory (Kotabe and Hofmann 2015). According to it, conflict between immediate desires and long-term goals signals the need to mobilize self-control resources. When self-control resources are abundant (control > desire), behaviors in line with long-term goals occur. However, when control resources are limited (control < desire), behaviors in line with immediate desires occur. Insofar as self-control attempts (i.e. incongruent Stroop trials) consume finite resources, they may tip the balance toward greater desire-driven reward-seeking behavior reflected in the enhanced RewP. Moreover, the results are consistent with theoretical models that conceptualize self-control as a value-based choice (Berkman et al. 2017; Pfeifer and Berkman, 2018). According to them, exercising control shifts value-based calculations in favor of more immediate options over (more effortful) options. Insofar as the RewP has been source localized to the striatum (Gehring and Willoughby 2002, Carlson et al. 2011, Foti et al. 2011, Becker et al. 2014) and the striatum tracks subjective value (Knutson et al. 2009), the finding of an enhanced RewP after self-control exertion may reflect shifting value-based calculations. Also, we note that the precise neural generators of the RewP remain uncertain (Cohen et al. 2011), and source localization of scalp-recorded ERPs is inherently challenging (Pizzagalli 2007).

Moreover, cognitive dissonance (Aronson and Mills 1959, Harmon-Jones and Mills 1999) and psychological contrast (Zentall 2010) accounts of effort suggest that aversive states elicited by the exertion of effort make the end-result or reward appear more valuable. In accord with these accounts, a greater subjective experience of effort is associated with a larger RewP in an effort justification paradigm (Harmon-Jones et al. 2020a), especially when perceptions of control are high (Harmon-Jones et al. 2024). To the extent that incongruent Stroop trials are effortful (Bouzidi and Gendolla 2023), the current results are consistent with effort-based interpretations of enhanced reward responsivity.

### Implications for ego-depletion and the strength model of self-control

The perspective advanced here adds conceptual and theoretical refinement to the resource modelm of self-control by identifying the specific circumstances under which exerting self-control influences subsequent behavior: increased reward responsivity. The resource model of self-control has been generative, making self-control research a focal point in social psychology for ∼25 years. However, this model has come under intense scrutiny and debate (Friese et al. 2019). Some researchers have suggested that the effects predicted by the resource model are smaller than once anticipated (Dang et al. 2017, Garrison et al. 2019), whereas others have suggested that these effects are negligible at best (Carter and McCullough 2014, Carter et al. 2015, MS et al. 2016, Vohs et al. 2021). By contrast, the original authors have reaffirmed their commitment to the model (Baumeister and Vohs 2016, Baumeister et al. 2018). The initial conceptualization of the resource model suggests that exercising self-control at Time 1 undermines the ability to exercise self-control at Time 2, resulting in decrement in performance on challenging tasks irrespective of task type. Stated otherwise, a domain-general, but finite, resource underlies all types of self-control (Baumeister et al. 1998, Muraven and Baumeister 2000). The current findings challenge the notion of domain generality and suggests that the effects of self-control exertion on subsequent behavior are specific to reward responsivity.

### Limitations and future directions

Although we interpreted the significant main effect of congruency on RewP amplitudes as supportive evidence of the reward responsivity hypothesis of self-control (Kelley et al. 2019), some readers may remain unconvinced due to the nonsignificant reward × congruency interaction. The RewP increases as a function of reward magnitude and even zero magnitude wins (Meadows et al. 2016, Threadgill and Gable 2018). Thus, the mere act of winning is rewarding even when it results in no monetary gain. Turning back to our findings, exercising self-control increases reward responsivity even to small (win £0) rewards, suggesting that exercising self-control produces domain general increases in reward responsivity. Nonetheless, the RewP difference wave results indicate that, relative to these small (win £0) rewards, participants were sensitive to hedonic and eudemonic rewards, a pattern consistent with a domain general increase in reward responsivity following self-control exertion. Nonetheless, the RewP is a complex marker of reward responsivity that tracks reward linking (Jia et al. 2013, Angus et al. 2015, Peterburs et al. 2019, Huvermann et al. 2021, Brown et al. 2022), reward wanting (Angus et al. 2015, Threadgill and Gable 2016, Huvermann et al. 2021, Banica et al. 2023), and reward learning (Cavanagh 2015, Jackson and Cavanagh 2023). Given this complexity, future studies are needed to more precisely characterize how exercising self-control modulates the multidimensionality of the RewP, thus providing a rigorous test of the reward responsivity hypothesis of self-control.

Multiple theoretical perspectives indicate that eudaimonic processes (e.g. meaning) are central to psychological experience (Frankl 1963, Becker 1971, Greenberg et al. 2004, Heine et al. 2006, Steger et al. 2008b, 2009, Wong 2013, Pyszczynski et al. 2015, Martela et al. 2018, Sedikides and Wildschut 2018). In an effort to maintain homeostasis, the impulses of the self often need to be held in check. These efforts (i.e. self-control exertion) often come at the cost of eudaimonic processes that gives life meaning, that is, autonomy, volition, and choice. Based on this theorizing and recent research (Campbell et al. 2024), we would have expected that self-control exertion produced stronger responses to eudaimonic over hedonic rewards. However, the RewP showed a domain general reward response. Still other researchers reported that the RewP to eudaimonic rewards is less sensitive to temporal decay than the RewP to hedonic rewards (Luo et al. 2022). In all, although self-control may not sensitize persons toward eudaimonic rewards in the moment, their weaker temporal decay may make eudaimonic rewards well suited for countermanding the aversiveness of self-control over time. Indeed, a weaker temporal decay of the RewP to eudaimonic (vs. hedonic) rewards may help to explain longitudinal associations between self-control and positive life outcomes (Moffitt et al. 2011). Still, other ERPs may be better suited to distinguish between hedonic and eudaimonic rewards after self-control exertion. For example, the late-positive potential is driven by stimulus significance above and beyond other factors (Hajcak and Foti 2020). Insofar as eudaimonic (vs. hedonic) rewards are more psychologically enriching, they should modulate the late-positive potential after self-control exertion. Future studies could test these possibilities.

## Conclusion

Self-control has profound implications for a wide range of behaviors, with grave personal and societal costs. Consequently, experimental research on self-control has permeated many subfields of psychology. Although challenges to prominent models have damped enthusiasm, we provided evidence supporting the reward responsivity hypothesis, with an increased reward responsivity (RewP) following self-control exertion. This effect occurs regardless of the presence or type of reward, suggesting that self-control enhances reward responsivity in a domain-general manner. We hope our findings offer the conceptual and theoretical innovation necessary to renew interest and focus to the experimental study of self-control.

## Supplementary Material

nsaf016_Supp

## References

[R1] Achtziger A, Alós-Ferrer C, Wagner AK. Money, depletion, and prosociality in the dictator game. *J Neurosci Psychol Econom* 2015;8:1–14. doi: 10.1037/npe0000031

[R2] Angus DJ, Kemkes K, Schutter DJLG et al. Anger is associated with reward‐related electrocortical activity: evidence from the reward positivity. *Psychophysiology* 2015;52:1271–80. doi: 10.1111/psyp.12460 26084980

[R3] Aronson E, Mills J. The effect of severity of initiation on liking for a group. *J Abnorm Soc Psychol* 1959;59:177–81. doi: 10.1037/h0047195

[R4] Banica I, Allison G, Racine SE et al. All the Pringle ladies: neural and behavioral responses to high‐calorie food rewards in young adult women. *Psychophysiology* 2023;60:e14188. doi: 10.1111/psyp.14188 36183246

[R5] Baumeister RF, Bratslavsky E, Muraven M et al. Ego depletion: is the active self a limited resource? *J Pers Soc Psychol* 1998;74:1252–65. doi: 10.1037/0022-3514.74.5.1252 9599441

[R6] Baumeister RF, Tice DM, Bushman BJ. A review of multisite replication projects in social psychology: is it viable to sustain any confidence in social psychology’s knowledge base? *Perspect Psychol Sci* 2023;18:912–35. doi: 10.1177/17456916221121815 36442681

[R7] Baumeister RF, Tice DM, Vohs KD. The strength model of self-regulation: conclusions from the second decade of willpower research. *Perspect Psychol Sci* 2018;13:141–45. doi: 10.1177/1745691617716946 29592652

[R8] Baumeister RF, Vohs KD. Misguided effort with elusive implications. *Perspect Psychol Sci* 2016;11:574–75. doi: 10.1177/1745691616652878 27474143

[R9] Baumeister RF, Vohs KD, Tice DM. The strength model of self-control. *Curr Dir Psychol Sci* 2007;16:351–55. doi: 10.1111/j.1467-8721.2007.00534.x

[R10] Becker E . *The Birth and Death of Meaning*. New York, NY: Free Press, 1971.

[R11] Becker MP, Nitsch AM, Miltner WH et al. A single-trial estimation of the feedback-related negativity and its relation to BOLD responses in a time-estimation task. *J Neurosci* 2014;34:3005–12. doi: 10.1523/JNEUROSCI.3684-13.2014 24553940 PMC6608516

[R12] Berkman ET, Hutcherson CA, Livingston JL et al. Self-Control as Value-Based Choice. *Curr Dir Psychol Sci* 2017:26;422–428. doi: 10.1177/0963721417704394 29335665 PMC5765996

[R13] Bogdanov M, Renault H, LoParco S et al. Cognitive effort exertion enhances electrophysiological responses to rewarding outcomes. *Cereb Cortex* 2022;32:4255–70. doi: 10.1093/cercor/bhab480 35169838

[R14] Bouzidi YS, Gendolla GH. Action‐orientation shields against primed cognitive conflict effects on effort‐related cardiac response. *Psychophysiology* 2023;60:e14407. doi: 10.1111/psyp.14407 37551961

[R15] Brown DR, Jackson TC, Cavanagh JF. The reward positivity is sensitive to affective liking. *Cogn Affect Behav Neurosci* 22 2022;258–267. doi: 10.3758/s13415-021-00950-5 34599487 PMC9578330

[R16] Bruyneel SD, Dewitte S, Franses PH et al. I felt low and my purse feels light: depleting mood regulation attempts affect risk decision making. *J Behav Decis Mak* 2009;22:153–70. doi: 10.1002/bdm.619

[R17] Cai H, Wu L, Shi Y et al. Self-enhancement among Westerners and Easterners: a cultural neuroscience approach. *Soc Cogn Affect Neurosci* 2016;11:1569–78. doi: 10.1093/scan/nsw072 27217110 PMC5040913

[R18] Campbell AV, Wang Y, Inzlicht M. Experimental evidence that exerting effort increases meaning. *PsyArXiv* 2024. doi: 10.31234/osf.io/z3g9c 39854968

[R19] Carlson JM, Foti D, Harmon-Jones E et al. Midbrain volume predicts fMRI and ERP measures of reward reactivity. *Brain Struct Funct* 2015;220:1861–66. doi: 10.1007/s00429-014-0725-9 24549705

[R20] Carlson JM, Foti D, Mujica-Parodi LR et al. Ventral striatal and medial prefrontal BOLD activation is correlated with reward-related electrocortical activity: a combined ERP and fMRI study. *NeuroImage* 2011;57:1608–16. doi: 10.1016/j.neuroimage.2011.05.037 21624476

[R21] Carter EC, Kofler LM, Forster DE et al. A series of meta-analytic tests of the depletion effect: self-control does not seem to rely on a limited resource. *J Exp Psychol Gen* 2015;144:796–815. doi: 10.1037/xge0000083 26076043

[R22] Carter EC, McCullough ME. Publication bias and the limited strength model of self-control: has the evidence for ego depletion been overestimated? *Front Psychol* 2014;5:823. doi: 10.3389/fpsyg.2014.00823 PMC4115664 25126083

[R23] Cavanagh JF . Cortical delta activity reflects reward prediction error and related behavioral adjustments, but at different times. *NeuroImage* 2015;110:205–16. doi: 10.1016/j.neuroimage.2015.02.007 25676913

[R24] Christiansen P, Cole JC, Field M. Ego depletion increases ad-lib alcohol consumption: investigating cognitive mediators and moderators. *Exp Clin Psychopharmacol* 2012;20:118–28. doi: 10.1037/a0026623 22182418

[R0024a] Cohen MX, Cavanagh JF, Slagter HA. Event-related potential activity in the basal ganglia differentiates rewards from nonrewards: Temporospatial principal components analysis and source localization of the feedback negativity: Commentary. *Human brain mapping* 2011;32:2270–2271. 21826758 10.1002/hbm.21358 PMC6870401

[R25] Dang J . Testing the role of glucose in self-control: a meta-analysis. *Appetite* 2016;107:222–30. doi: 10.1016/j.appet.2016.07.021 27492453

[R26] Dang J, Liu Y, Liu X et al.. The ego could be depleted, providing initial exertion is depleting. *Soc Psychol* 2017;48:242–45. doi: 10.1027/1864-9335/a000308

[R27] David L, Vassena E, Bijleveld E. The unpleasantness of thinking: a meta-analytic review of the association between mental effort and negative affect. *Psychol Bull* 2024;150:1070–93. doi: 10.1037/bul0000443 39101924

[R28] Delorme A, Makeig S. EEGLAB: an open source toolbox for analysis of single-trial EEG dynamics including independent component analysis. *J Neurosci Methods* 2004;134:9–21. doi: 10.1016/j.jneumeth.2003.10.009 15102499

[R29] Disabato DJ, Goodman FR, Kashdan TB et al. Different types of well-being? A cross-cultural examination of hedonic and eudaimonic well-being. *Psychol Assess* 2016;28:471–82. doi: 10.1037/pas0000209 26348031

[R30] Faul F, Erdfelder E, Buchner A et al. Statistical power analyses using G*Power 3.1: tests for correlation and regression analyses. *Behav Res Methods* 2009;41:1149–60. doi: 10.3758/BRM.41.4.1149 19897823

[R31] Finley AJ, Schmeichel BJ. Aftereffects of self-control on positive emotional reactivity. *Pers Soc Psychol Bull* 2019;45:1011–27. doi: 10.1177/0146167218802836 30400747

[R32] Foti D, Carlson JM, Sauder CL et al. Reward dysfunction in major depression: multimodal neuroimaging evidence for refining the melancholic phenotype. *NeuroImage* 2014;101:50–58. doi: 10.1016/j.neuroimage.2014.06.058 24996119 PMC4165813

[R33] Foti D, Weinberg A, Dien J et al. Event‐related potential activity in the basal ganglia differentiates rewards from nonrewards: temporospatial principal components analysis and source localization of the feedback negativity. *Hum Brain Mapp* 2011;32:2207–16. doi: 10.1002/hbm.21182 21305664 PMC6870417

[R34] Frankl VE . *Man’s Search for Meaning*. New York, NY: Washington Square Press, 1963.

[R35] Friese M, Loschelder DD, Gieseler K et al. Is ego depletion real? An analysis of arguments. *Pers Soc Psychol Rev* 2019;23:107–31. doi: 10.1177/1088868318762183 29591537

[R36] Gailliot MT, Baumeister RF, DeWall CN et al. Self-control relies on glucose as a limited energy source: willpower is more than a metaphor. *J Pers Soc Psychol* 2007;92:325–36. doi: 10.1037/0022-3514.92.2.325 17279852

[R37] Gallagher MW, Lopez SJ, Preacher KJ. The hierarchical structure of well‐being. *J Pers* 2009;77:1025–50. doi: 10.1111/j.1467-6494.2009.00573.x 19558444 PMC3865980

[R38] Garrison KE, Finley AJ, Schmeichel BJ. Ego depletion reduces attention control: evidence from two high-powered preregistered experiments. *Pers Soc Psychol Bull* 2019;45:728–39. doi: 10.1177/0146167218796473 30239268

[R39] Gehring WJ, Willoughby AR. The medial frontal cortex and the rapid processing of monetary gains and losses. *Science* 2002;295:2279–82. doi: 10.1126/science.1066893 11910116

[R40] Gendolla GHE Wright RA . Effort. In: Sander D and Scherer KR (eds), *Oxford Companion to Emotion and the Affective Sciences*. Oxford: Oxford University Press, 2009, 134–35.

[R41] Glazer JE, Kelley NJ, Pornpattananangkul N et al. Beyond the FRN: broadening the time-course of EEG and ERP components implicated in reward processing. *Int J Psychophysiol* 2018;132:184–202. doi: 10.1016/j.ijpsycho.2018.02.002 29454641

[R42] Goodman FR, Disabato DJ, Kashdan TB et al. Measuring well-being: a comparison of subjective well-being and PERMA. *J Posit Psychol* 2018;13:321–32. doi: 10.1080/17439760.2017.1388434

[R43] Greenberg J, Koole SL and Pyszczynski TA. (eds). *Handbook of Experimental Existential Psychology*. New York, NK: Guilford Press, 2004

[R44] Hagger MS, Wood C, Stiff C et al. Ego depletion and the strength model of self-control: a meta-analysis. *Psychological Bulletin* 2010;136:495–525. doi: 10.1037/a0019486 20565167

[R45] Hajcak G, Foti D. Significance? Significance! Empirical, methodological, and theoretical connections between the late positive potential and P300 as neural responses to stimulus significance: an integrative review. *Psychophysiology* 2020;57:e13570. doi: 10.1111/psyp.13570 32243623

[R46] Harmon-Jones E, Clarke D, Paul K et al. The effect of perceived effort on reward valuation: taking the reward positivity (RewP) to dissonance theory. *Front Hum Neurosci* 2020a;14:157. doi: 10.3389/fnhum.2020.00157 PMC7241252 32477082

[R47] Harmon-Jones E, Matis S, Angus DJ et al. Does effort increase or decrease reward valuation? Considerations from cognitive dissonance theory. *Psychophysiology* 2024;61:e14536. doi: 10.1111/psyp.14536 38323360

[R48] Harmon-Jones E, Mills J. *Cognitive Dissonance: Progress on a Pivotal Theory in Social Psychology*. Washington, DC: American Psychological Association, 1999.

[R49] Harmon-Jones E, Willoughby C, Paul K et al. The effect of perceived effort and perceived control on reward valuation: using the reward positivity to test a dissonance theory prediction. *Biol Psychol* 2020b;154:107910. doi: 10.1016/j.biopsycho.2020.107910 32473260

[R50] Haynes A, Kemps E, Moffitt R. Too depleted to try? Testing the process model of ego depletion in the context of unhealthy snack consumption. *Appl Psychol Health Well‐Being* 2016;8:386–404. doi: 10.1111/aphw.12080 27758050

[R51] Heine SJ, Proulx T, Vohs KD. The meaning maintenance model: on the coherence of social motivations. *Pers Soc Psychol Rev* 2006;10:88–110. doi: 10.1207/s15327957pspr1002_1 16768649

[R52] Henderson LW, Knight T, Richardson B. An exploration of the well-being benefits of hedonic and eudaimonic behaviour. *J Posit Psychol* 2013;8:322–36. doi: 10.1080/17439760.2013.803596

[R53] Hofmann W, Rauch W, Gawronski B. And deplete us not into temptation: automatic attitudes, dietary restraint, and self-regulatory resources as determinants of eating behavior. *J Exp Soc Psychol* 2007;43:497–504. doi: 10.1016/j.jesp.2006.05.004

[R54] Holroyd CB, Hajcak G, Larsen JT. The good, the bad and the neutral: electrophysiological responses to feedback stimuli. *Brain Res* 2006;1105:93–101. doi: 10.1016/j.brainres.2005.12.015 16427615

[R55] Holroyd CB, Krigolson OE, Lee S. Reward positivity elicited by predictive cues. *NeuroReport* 2011;22:249–52. doi: 10.1097/WNR.0b013e328345441d 21386699

[R56] Holroyd CB, Pakzad‐Vaezi KL, Krigolson OE. The feedback correct‐related positivity: sensitivity of the event‐related brain potential to unexpected positive feedback. *Psychophysiology* 2008;45:688–97. doi: 10.1111/j.1469-8986.2008.00668.x 18513364

[R57] Huta V, Waterman AS. Eudaimonia and its distinction from hedonia: developing a classification and terminology for understanding conceptual and operational definitions. *J Happ Stud* 2014;15:1425–56. doi: 10.1007/s10902-013-9485-0

[R58] Huvermann DM, Bellebaum C, Peterburs J. Selective devaluation affects the processing of preferred rewards. *Cogn Affect Behav Neurosci* 2021;21:1010–25. doi: 10.3758/s13415-021-00904-x 33931831 PMC8455391

[R59] Imhoff R, Schmidt AF, Gerstenberg F. Exploring the interplay of trait self-control and ego depletion: empirical evidence for ironic effects. *Eur J Pers* 2014;28:413–24. doi: 10.1002/per.1899

[R60] Inzlicht M, Schmeichel B. What is ego depletion? Toward a mechanistic revision of the resource model of self-control. *Perspect Psychol Sci* 2012;7:450–63. doi: 10.1177/1745691612454134 26168503

[R61] Inzlicht M, Schmeichel BJ, Macrae CN. Why self-control seems (but may not be) limited. *Trends Cogn Sci* 2014;18:127–33. doi: 10.1016/j.tics.2013.12.009 24439530

[R62] Jackson TC, Cavanagh JF. Reduced positive affect alters reward learning via reduced information encoding in the Reward Positivity. *Psychophysiology* 2023;60:e14276. doi: 10.1111/psyp.14276 36807324

[R63] Jia S, Zhang W, Li P et al. Attitude toward money modulates outcome processing: an ERP study. *Soc Neurosci* 2013;8:43–51. doi: 10.1080/17470919.2012.713316 22856426

[R64] Joshanloo M . Revisiting the empirical distinction between hedonic and eudaimonic aspects of well-being using exploratory structural equation modeling. *J Happ Stud* 2016;17:2023–36. doi: 10.1007/s10902-015-9683-z

[R65] Kashdan TB, Biswas-Diener R, King LA. Reconsidering happiness: the costs of distinguishing between hedonics and eudaimonia. *J Posit Psychol* 2008;3:219–33. doi: 10.1080/17439760802303044

[R66] Kelley NJ, Finley AJ, Schmeichel BJ. After-effects of self-control: the reward responsivity hypothesis. *Cogn Affect Behav Neurosci* 2019;19:600–18. doi: 10.3758/s13415-019-00694-3 30673962 PMC8182659

[R67] Knutson B, Adams CM, Fong GW et al. Anticipation of increasing monetary reward selectively recruits nucleus accumbens. *J Neurosci* 2001;21:RC159. doi: 10.1523/JNEUROSCI.21-16-j0002.2001 PMC6763187 11459880

[R68] Knutson B Delgado MR Phillips PE . Representation of subjective value in the striatum. In: Glimcher PW, Camerer CF and Fehr E, et al. (eds), *Neuroeconomics: Decision Making and the Brain*. New York, NY: Academic Press, 2009, 389–406

[R69] Kotabe HP, Hofmann W. On integrating the components of self-control. *Perspect Psychol Sci* 2015;10:618–38. doi: 10.1177/1745691615593382 26386000

[R70] Kurzban R . The sense of effort. *Curr Opin Psychol* 2016;7:67–70. doi: 10.1016/j.copsyc.2015.08.003

[R71] Lange S, Leue A, Beauducel A. Behavioral approach and reward processing: results on feedback-related negativity and P3 component. *Biol Psychol* 2012;89:416–25. doi: 10.1016/j.biopsycho.2011.12.004 22178442

[R72] Liu X, Hairston J, Schrier M et al. Common and distinct networks underlying reward valence and processing stages: a meta-analysis of functional neuroimaging studies. *Neurosci Biobehav Rev* 2011;35:1219–36. doi: 10.1016/j.neubiorev.2010.12.012 21185861 PMC3395003

[R73] Luck SJ, Gaspelin N. How to get statistically significant effects in any ERP experiment (and why you shouldn’t). *Psychophysiology* 2017;54:146–57. doi: 10.1111/psyp.12639 28000253 PMC5178877

[R74] Luo Y, Jiang H, Chen X et al. Temporal dynamics of hedonic and eudaimonic reward processing: an event-related potentials (ERPs) study. *Int J Psychophysiol* 2019;137:63–71. doi: 10.1016/j.ijpsycho.2018.12.009 30576767

[R75] Luo Y, Zhang X, Jiang H et al. The neural habituation to hedonic and eudaimonic rewards: evidence from reward positivity. *Psychophysiology* 2022;59:e13977. doi: 10.1111/psyp.13977 34846754

[R76] Ma Q, Meng L, Wang L et al.. I endeavor to make it: effort increases valuation of subsequent monetary reward. *Behav Brain Res* 2014;261:1–7. doi: 10.1016/j.bbr.2013.11.045 24308956

[R77] Martela F, Ryan RM, Steger MF. Meaningfulness as satisfaction of autonomy, competence, relatedness, and beneficence: comparing the four satisfactions and positive affect as predictors of meaning in life. *J Happ Stud* 2018;19:1261–82. doi: 10.1007/s10902-017-9869-7

[R78] Meadows CC, Gable PA, Lohse KR et al. The effects of reward magnitude on reward processing: an averaged and single trial event-related potential study. *Biol Psychol* 2016;118:154–60. doi: 10.1016/j.biopsycho.2016.06.002 27288743

[R79] Miltner WH, Braun CH, Coles MG. Event-related brain potentials following incorrect feedback in a time-estimation task: evidence for a “generic” neural system for error detection. *J Cogn Neurosci* 1997;9:788–98. doi: 10.1162/jocn.1997.9.6.788 23964600

[R80] Moffitt TE, Arseneault L, Belsky D et al. A gradient of childhood self-control predicts health, wealth, and public safety. *Proc Natl Acad Sci* 2011;108:2693–98. doi: 10.1073/pnas.1010076108 21262822 PMC3041102

[R81] Morelli SA, Sacchet MD, Zaki J. Common and distinct neural correlates of personal and vicarious reward: a quantitative meta-analysis. *NeuroImage* 2015;112:244–53. doi: 10.1016/j.neuroimage.2014.12.056 25554428 PMC4408229

[R82] MS H, NL C, Alberts H et al. A multilab preregistered replication of the ego-depletion effect. *Perspect Psychol Sci* 2016;11:546–73. doi: 10.1177/1745691616652873 27474142

[R83] Muraven M, Baumeister RF. Self-regulation and depletion of limited resources: does self-control resemble a muscle? *Psychol Bull* 2000;126:247–59. doi: 10.1037/0033-2909.126.2.247 10748642

[R84] Muraven M, Collins RL, Neinhaus K. Self-control and alcohol restraint: an initial application of the self-control strength model. *Psychol Addic Behav* 2002;16:113–20. doi: 10.1037/0893-164X.16.2.113 12079249

[R85] Muraven M, Shmueli D, Burkley E. Conserving self-control strength. *J Pers Soc Psychol* 2006;91:524–37. doi: 10.1037/0022-3514.91.3.524 16938035

[R86] Muraven M, Tice DM, Baumeister RF. Self-control as a limited resource: regulatory depletion patterns. *J Pers Soc Psychol* 1998;74:774–89. doi: 10.1037/0022-3514.74.3.774 9523419

[R87] Osgood JM, Muraven M. Self-control depletion does not diminish attitudes about being prosocial but does diminish prosocial behaviors. *Basic Appl Soc Psychol* 2015;37:68–80. doi: 10.1080/01973533.2014.996225

[R88] Pan W, Lu J, Wu L et al. Expending effort may share neural responses with reward and evokes high subjective satisfaction. *Biol Psychol* 2023;177:108480. doi: 10.1016/j.biopsycho.2022.108480 36603735

[R89] Peirce JW . PsychoPy—Psychophysics software in Python. *J Neurosc Methods* 2007;162:8–13. doi: 10.1016/j.jneumeth.2006.11.017 PMC2018741 17254636

[R90] Perrin F, Pernier J, Bertrand O et al. Spherical splines for scalp potential and current density mapping. *Electroencephalogr Clin Neurophysiol* 1989;72:184–87. doi: 10.1016/0013-4694(89)90180-6 2464490

[R91] Peterburs J, Sannemann L, Bellebaum C. Subjective preferences differentially modulate the processing of rewards gained by own vs. observed choices. *Neuropsychologia* 2019;132:107–39. doi: 10.1016/j.neuropsychologia.2019.107139 31295450

[R92] Pfeifer JH, Berkman ET. The Development of Self and Identity in Adolescence: Neural Evidence and Implications for a Value‐Based Choice Perspective on Motivated Behavior *Child Dev Perspectives* 2018;12:158–164. doi: 10.1111/cdep.12279 PMC6667174 31363361

[R93] Pion-Tonachini L, Kreutz-Delgado K, Makeig S. ICLabel: an automated electroencephalographic independent component classifier, dataset, and website. *NeuroImage* 2019;198:181–97. doi: 10.1016/j.neuroimage.2019.05.026 31103785 PMC6592775

[R94] Pizzagalli DA . Electroencephalography and high-density electrophysiology source localization. In: Cacioppo JT, Tassinary LG and Berntson GG (eds), *Handbook of Psychophysiology*. Cambridge: Cambridge University Press, 2007, 56–84

[R95] Pyszczynski T, Solomon S, Greenberg J. Thirty years of terror management theory: from genesis to revelation. *Adv Exp Soc Psychol* 2015;52:1–70. doi: 10.1016/bs.aesp.2015.03.001

[R96] Ryan RM, Deci EL. On happiness and human potentials: a review of research on hedonic and eudaimonic well-being. *Annu Rev Psychol* 2001;52:118–28. doi: 10.1037/a0026623 11148302

[R97] San Martín R, Kwak Y, Pearson JM et al. Altruistic traits are predicted by neural responses to monetary outcomes for self vs charity. *Soc Cogn Affect Neurosci* 2016;11:863–76. doi: 10.1093/scan/nsw026 27030510 PMC4884320

[R98] Schlauch RC, Christensen RL, Derrick JL et al. Individual differences in approach and avoidance inclinations moderate the effect of self-control depletion on ad-lib drinking. *Alcohol Clin Exp Res* 2015;39:2480–88. doi: 10.1111/acer.12915 26756800 PMC4710858

[R99] Schmeichel BJ, Harmon-Jones C, Harmon-Jones E. Exercising self-control increases approach motivation. *J Pers Soc Psychol* 2010;99:162–73. doi: 10.1037/a0019797 20565193

[R100] Sedikides C, Wildschut T. Finding meaning in nostalgia. *Rev Gen Psychol* 2018;22:48–61. doi: 10.1037/gpr0000109

[R101] Sescousse G, Caldú X, Segura B et al. Processing of primary and secondary rewards: a quantitative meta-analysis and review of human functional neuroimaging studies. *Neurosci Biobehav Rev* 2013;37:681–96. doi: 10.1016/j.neubiorev.2013.02.002 23415703

[R102] Shizgal P . On the neural computation of utility: implications from studies of brain stimulation reward. In: Kahneman D, Diener E and Schwarz N (eds), *Well-being: The Foundations of Hedonic Psychology*. New York, NY: Russell Sage Foundation, 1999, 502–26

[R103] Shmueli D, Prochaska JJ. Resisting tempting foods and smoking behavior: implications from a self-control theory perspective. *Health Psychol* 2009;28:300–06. doi: 10.1037/a0013826 19450035 PMC2736876

[R104] Smith T, Panfil K, Bailey C et al. Cognitive and behavioral training interventions to promote self-control. *J Exp Psychol Anim Learn Cogn* 2019;45:259–79. doi: 10.1037/xan0000208 31070430 PMC6716382

[R105] Steger MF, Kashdan TB, Oishi S. Being good by doing good: daily eudaimonic activity and well-being. *J Res Pers* 2008a;42:22–42. doi: 10.1016/j.jrp.2007.03.004

[R106] Steger MF, Kashdan TB, Sullivan BA et al. Understanding the search for meaning in life: personality, cognitive style, and the dynamic between seeking and experiencing meaning. *J Pers* 2008b;76:199–228. doi: 10.1111/j.1467-6494.2007.00484.x 18331281

[R107] Steger MF, Oishi S, Kashdan TB. Meaning in life across the life span: levels and correlates of meaning in life from emerging adulthood to older adulthood. *J Posit Psychol* 2009;4:43–52. doi: 10.1080/17439760802303127

[R108] Telzer EH, Fuligni AJ, Lieberman MD et al. Neural sensitivity to eudaimonic and hedonic rewards differentially predict adolescent depressive symptoms over time. *Proc Natl Acad Sci* 2014;111:6600–05. doi: 10.1073/pnas.1323014111 24753574 PMC4020059

[R109] Threadgill AH, Gable PA. Approach-motivated pregoal states enhance the reward positivity. *Psychophysiology* 2016;53:733–38. doi: 10.1111/psyp.12611 26799161

[R110] Threadgill AH, Gable PA. The sweetness of successful goal pursuit: approach-motivated pregoal states enhance the reward positivity during goal pursuit. *Int J Psychophysiol* 2018;132:277–86. doi: 10.1016/j.ijpsycho.2017.12.010 29274365

[R111] Vohs K, Baumeister R, Mead N et al. Engaging in self-control intensifies desires and feelings. In:. *Building connections: Proceedings of the Association for Consumer Research Annual Conference* Duluth, MN. Association for Consumer Research, 2011

[R112] Vohs KD, Baumeister RF. *Handbook of Self-regulation: Research, Theory, and Applications*. New York, NY: Guilford Press, 2016

[R113] Vohs KD, Schmeichel BJ, Lohmann S et al. A multisite preregistered paradigmatic test of the ego-depletion effect. *Psychol Sci* 2021;32:1566–81. doi: 10.1177/0956797621989733 34520296 PMC12422598

[R114] Walsh MM, Anderson JR. Learning from experience: event-related potential correlates of reward processing, neural adaptation, and behavioral choice. *Neurosci Biobehav Rev* 2012;36:1870–84. doi: 10.1016/j.neubiorev.2012.05.008 22683741 PMC3432149

[R115] Wong PT . *The Human Quest for Meaning: Theories, Research, and Applications*. New York, NY: Routledge, 2013

[R116] Zentall TR . Justification of effort by humans and pigeons: cognitive dissonance or contrast? *Curr Dir Psychol Sci* 2010;19:196–300. doi: 10.1177/0963721410383381

[R117] Zhang Y, Rong Y, Wei P. Mothers exhibit higher neural activity in gaining rewards for their children than for themselves. *Soc Cogn Affect Neurosci* 2023;18:1–14. doi: 10.1093/scan/nsad048 PMC10558201 37702293

