# Supplementary material for: Exercising self-control increases responsivity to hedonic and eudaimonic rewards
Source: Soc Cogn Affect Neurosci. 2025 Jan 30;20(1):nsaf016. doi: 10.1093/scan/nsaf016 (PMC11817797; doi:10.1093/scan/nsaf016)
Supplement: nsaf016_Supp [file nsaf016_supp.zip › scan-24-239-File007.docx]

**Figure S1**

*Grand Averages of RewP Amplitude as a Function of Reward and Congruency*


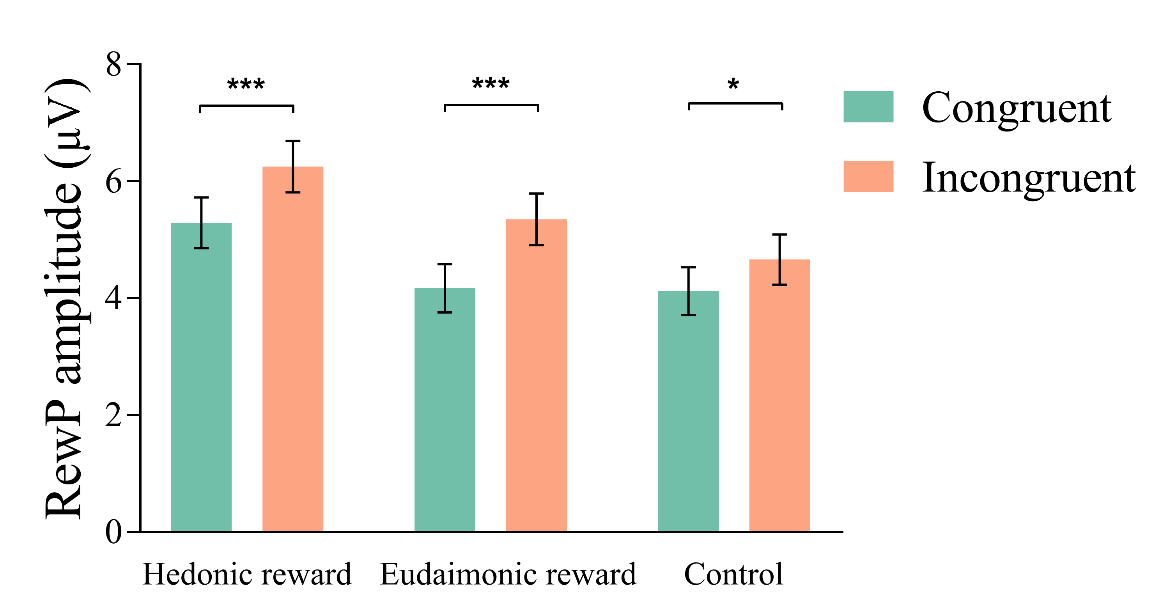


*Note.* Error bars represent SEM; ^***^*p* < .001. ^**^*p* < .01. ^*^*p* < .05.

**Figure S2**

*Grand Averages of RewP Difference Wave as a Function of Reward and Congruency*


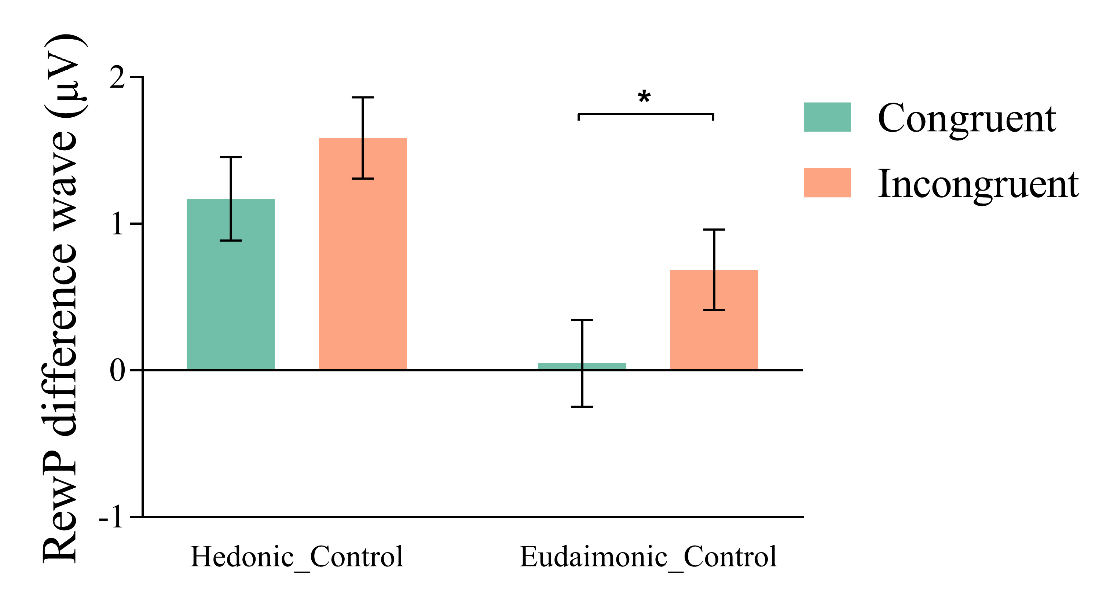


*Note.* Error bars represent SEM; ^*^*p* < .05.
